# Venetoclax with Decitabine or Azacitidine in Relapsed or Refractory Acute Myeloid Leukemia

**DOI:** 10.21203/rs.3.rs-3015916/v1

**Published:** 2023-06-12

**Authors:** Ian M. Bouligny, Graeme Murray, Thuy Ho, Michael Doyel, Tilak Patel, Josh Boron, Valerie Tran, Juhi Gor, Yiwei Hang, Yanal Alnimer, Kyle Zacholski, Chad Venn, Nolan A. Wages, Steven Grant, Keri R. Maher

**Affiliations:** 1 Virginia Commonwealth University Massey Cancer Center — NCI Designated Comprehensive Cancer Center, Division of Hematology and Oncology, Department of Internal Medicine, 1001 E. Leigh St., Richmond, VA, USA; 2 Virginia Commonwealth University School of Medicine, 1201 E. Marshall St., Richmond, VA, USA; 3 Virginia Commonwealth University Medical Center, Department of Internal Medicine, 1101 E. Marshall St., Richmond, VA, USA; 4 Virginia Commonwealth University Medical Center, Department of Pharmacy, 410 North 12^th^ St., Richmond, VA, USA; 5 Virginia Commonwealth University School of Medicine, Department of Biostatistics, 830 E. Main St., Richmond, VA, USA

**Keywords:** AML, venetoclax, relapsed, refractory, hypomethylating agent

## 1. Introduction

Relapsed or refractory AML carries a dismal prognosis [1]. The introduction of venetoclax, a BCL-2 antagonist, in combination with a hypomethylating agent or low-dose cytarabine provided a novel augmented treatment approach — particularly for older adults and those ineligible for intensive induction [2]. Consequently, much work is now focused on identifying subgroups of disease that either respond favorably or are resistant to lower-intensity venetoclax-based strategies.

**Fig. 1:**
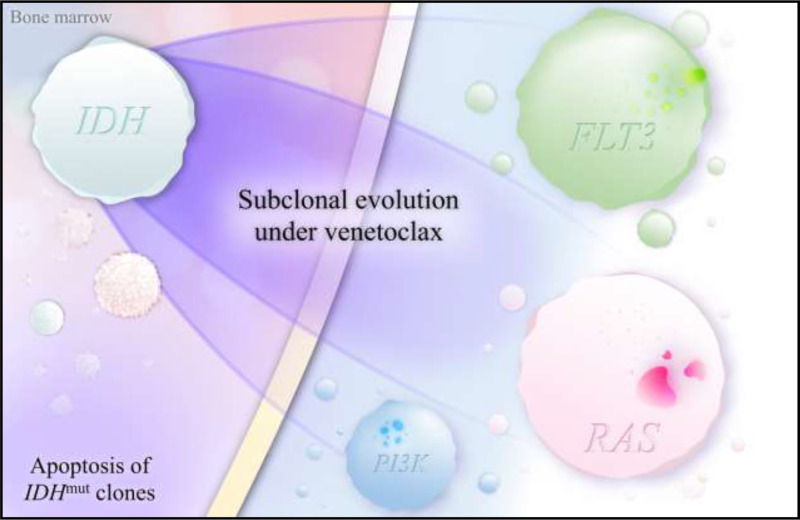
Subclonal evolution and adaptive resistance under selective pressure with venetoclax. Created with Pixelmator Pro.

The European LeukemiaNet (ELN) 2022 guidelines refined the molecular stratification schema for patients with AML, resulting in improved prognoses for those treated with intensive chemotherapy [3]. However, outcomes need to be clarified in the context of lower-intensity venetoclax-based strategies. Our group, among others, reported significant survival differences in unique molecular cohorts unaccounted for in ELN 2022, such as AML with mutated *IDH1* or *IDH2* [4–7]. Additionally, preclinical studies and updated analyses of clinical trials have suggested that signaling mutations are implicated in primary refractoriness or adaptive resistance to venetoclax — including mutations in *FLT3*, *PI3K*, and *RAS*, as shown in Figure 1 [5, 8–10]. The impact of these mutations is unknown in the context of relapsed or refractory AML treated with venetoclax-based strategies.

Few retrospective studies have examined the molecular impact of venetoclax in the relapsed or refractory setting [11]; even fewer clinical trials investigated the outcomes of venetoclax and a hypomethylating agent in relation to unique molecular aberrations [5]. Furthermore, the selection of therapeutic candidates outside clinical trials needs to be elucidated. While the VIALE-A trial — which investigated venetoclax and azacitidine versus azacitidine alone — included older adults and those ineligible for intensive induction in the front-line setting, there is little robust data examining outcomes with respect to performance status in the relapsed or refractory setting [2]. Consequently, clinical data remain sparse for the optimal selection of therapeutic candidates for lower-intensity venetoclax-based salvage therapy.

Additionally, the introduction of new therapies in the treatment of AML has influenced the sequencing of therapeutic options in the relapsed or refractory setting. The FDA approval of FLT3 and IDH inhibitors in the treatment of relapsed or refractory AML has prompted several new questions [12–14]. Accordingly, clinicians often find themselves choosing between novel therapies or intensive salvage strategies after venetoclax failure, despite the paucity of reports illuminating outcomes in this setting. Analyses of therapeutic options are sorely needed for patients who have failed venetoclax-based salvage therapy.

## 2. Methods

### 2.1 Objectives

There were two primary objectives of this study: to determine the composite complete remission rate and overall survival of patients with relapsed or refractory AML treated with venetoclax and decitabine or azacitidine. The secondary objectives were to characterize toxicities associated with venetoclax and decitabine or azacitidine, determine patient and disease-related predictors of survival, and investigate the response and survival associated with regimens following venetoclax failure.

### 2.2 Patient Eligibility

The Institutional Review Board of Virginia Commonwealth University Medical Center approved this single-center, retrospective study involving 57 consecutive patients analyzed over 204 treatment phases. Eligibility criteria included all patients aged 18 years or older with relapsed or refractory AML that received at least one dose of venetoclax with decitabine or azacitidine from January 2018 to January 2022. Patients who received venetoclax with a hypomethylating agent for a prior line of therapy were excluded. Patients were excluded if death occurred before the first dose of disease-directed therapy or if treatment records were unavailable for retrospective analysis. We captured patient fitness using the Charlson comorbidity index (CCI) score at diagnosis and the ECOG score at the start of each treatment phase [15].

### 2.3. Treatment Regimens

Patients received venetoclax on day one of treatment and continued until the end of the 28-day cycle or shorter duration, adjusted for toxicity or drug-drug interactions. Venetoclax was administered in 28-day cycles with decitabine 20 mg/m^2^ in 5- or 10-day courses or azacitidine 75 mg/m^2^ in 5- or 7-day courses. Venetoclax and decitabine or azacitidine were administered as maintenance in 28-day cycles until intolerability, disease progression, or death, with cycle delays and dose adjustments allowed for adverse events or count recovery.

### 2.4 Data Collection and Entry

We designed a REDCap instrument to retrospectively capture patient data [16]. The instrument was programmed to include cytogenetic and molecular profiles, response, and toxicity for each phase of treatment, including induction, maintenance, and relapse. Built-in score calculators and survival computation were programmed into the instrument during development to standardize data entry among investigators and minimize the likelihood of analytical errors.

The lead investigator reviewed the data set at three pre-specified time points and cross-checked entries for accuracy with the electronic medical record (Cerner Millennium and Epic). A minimum of two investigators standardized and cross-checked response and toxicity grading. At the conclusion of data collection, the lead investigator resolved flagged data discrepancies in a third review.

### 2.4 Safety Analysis

Toxicities were graded using the Common Terminology Criteria for Adverse Events (CTCAE) version 5.0 [17, 18]. Treatment-related adverse events were included if they occurred between the first dose and 28 days following treatment discontinuation. Quantitative toxicities were graded and recorded throughout each patient’s treatment phase, excluding electrolyte abnormalities. In instances where complete records were unavailable, toxicities were marked as unavailable for the phase of treatment to reduce bias.

### 2.5 Cytogenetic and Molecular Analysis

AML was defined using the fourth edition World Health Organization criteria, with a minimum of one bone marrow biopsy demonstrating at least 20% or greater myeloblasts [19]. The genetic risk was defined as recommended by the European LeukemiaNet 2022 guidelines [3]. PCR assays obtained at diagnosis had a sensitivity of 10^−4^ for *NPM1* and 10^−2^ for *CEBPA*, *FLT3*-ITD, and *FLT3*-TKD. Next-generation sequencing (NGS) was performed using an in-house NGS assay with a sensitivity of 2.7 × 10^−2^.

MRD negativity was defined using an assay at a minimum sensitivity threshold of 10^−3^, including PCR-based MRD assays and multiparameter flow cytometry (MFC; University of Washington Medical Center). Mutations frequently associated with clonal hematopoiesis, including *DNMT3A*, *TET2*, and *ASXL1*, were not considered MRD if detected on a remission NGS assay [20]. Similarly, germline mutations, such as *DDX41*, *GATA2*, and *TP53*, were excluded as MRD [20]. MRD-negative results with suboptimal sample quality, as indicated in the result report, were excluded from MRD analysis.

### 2.6 Response Assessment

Response assessments were performed in accordance with the modified International Working Group response criteria for AML [21]. Complete remission (CR) was defined as an absolute neutrophil count (ANC) greater than 1.0 × 10^9^/L, a platelet count greater than 100 × 10^9^/L, transfusion independence, and a bone marrow biopsy with less than 5% blasts. CR with incomplete hematologic recovery (CRi) was defined as all the criteria for CR except for neutropenia (ANC ≤1.0 × 10^9^/L) or thrombocytopenia (platelets ≤100 × 10^9^/L). CR with partial hematologic recovery (CRh) was defined as all the criteria for CR except for lower ANC (>0.5 × 10^9^/L) and platelet (>50 × 10^9^/L) thresholds. Morphological leukemia-free state (MLFS) was defined as less than 5% blasts and not meeting any of the above criteria for count recovery. Progressive disease was defined as outlined by the European LeukemiaNet guidelines [22]. Composite complete remission (CRc) rates included patients that achieved CR, CRi, or CRh.

### 2.7 Statistical Analysis

Patients treated between January 1, 2018 and January 1, 2022 were included in the study. The clinical data cutoff date was August 1, 2022, and patients alive at that time were censored. Means between groups were compared using the nonparametric Mann-Whitney test. Remission rates were reported and used the Wilson method for 95% confidence intervals; between-group comparisons used Fisher’s exact test. The overall survival was estimated for each cohort using the Kaplan-Meier method and compared using the log-rank test. All reported *p*-values were two-sided, with statistical significance evaluated at the 0.05 alpha level. Data analysis was performed with GraphPad Prism 9.4.1 for Macintosh.

## 3. Results

### 3.1 Demographics and Baseline Characteristics

We identified 57 patients with relapsed or refractory AML treated with venetoclax and decitabine or azacitidine. The median age at diagnosis was 60 years (range, 23 – 81 y.). Twenty-eight (49.1%) were male, and 76.8% identified as White. Patients were classified by ELN 2022 risk stratification: eight (14.0%) were favorable risk, 12 (21.1%) were intermediate risk, and 37 (64.9%) were adverse risk. The most common mutations were *NPM1* (23.6%), *NRAS* (21.8%), and *FLT3*-ITD (20.0%). Twelve (22.6%) patients had diagnostic marrow samples consistent with AML with myelodysplasia-related changes (AML-MRC), and four (7.0%) patients had an antecedent myelodysplastic neoplasm (MDS).

We then assessed patient fitness at the time of diagnosis. The median CCI score was 4 (range, 2 – 14), and the median ECOG score was 1 (range, 0 – 3). We observed treatment strategies prior to salvage with venetoclax and a hypomethylating agent; 8 (14.0%) patients received an allogeneic stem cell transplant prior to venetoclax exposure, and 11 (19.3%) patients were previously exposed to a hypomethylating agent.

At the initiation of venetoclax-based salvage, 24 (42.1%) patients had relapsed disease, and the remainder (57.9%) had refractory disease to one or more lines of therapy. The overall cohort underwent a median of one prior line of therapy (range, 1 – 7). Patients received a median of two (range, 1 – 10) cycles of venetoclax and a hypomethylating agent. The baseline demographics of the study population are detailed in Table 1.

### 3.2 Toxicity

**Fig. 2:**
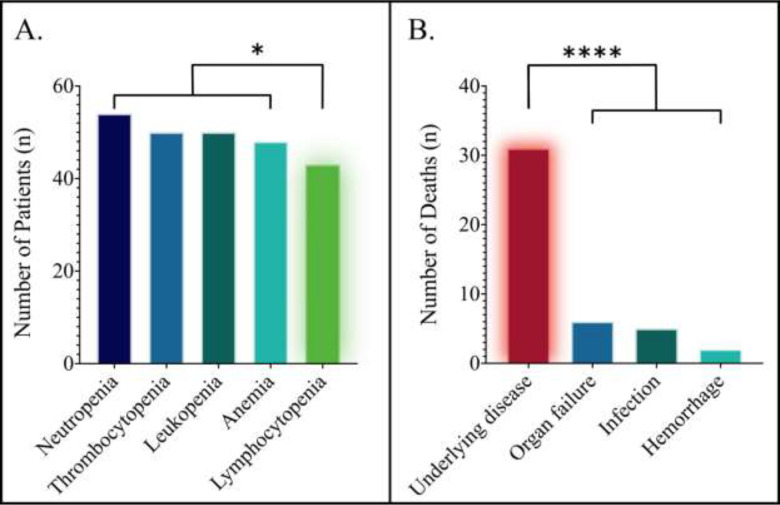
Toxicities associated with venetoclax and a hypomethylating agent in relapsed or refractory AML. A. Frequencies of high-grade hematological toxicity; significantly fewer patients had grade 3 or higher lymphocytopenia compared with the remaining hematological toxicities (*p* = 0.018). B. Causes of death in relapsed or refractory AML treated with venetoclax and a hypomethylating agent; death from underlying disease occurred significantly more frequently than any other cause (*p* < 0.0001)

Fifty-three of 57 patients had toxicity data available for analysis; the frequencies of toxicities across all cycles are presented in Table 2. Two patients from the decitabine and azacitidine cohorts switched the hypomethylating backbone during maintenance, providing a total of 55 treatment courses evaluable for toxicity. The most common grade three or higher toxicities were hematologic. The most frequent grade three or higher hematologic toxicity was neutropenia, which occurred in 54 (98.2%) patients. There was a significantly lower rate of lymphocytopenia compared with the remaining hematological toxicities (*p* = 0.018), depicted in Figure 2A. There were no significant differences in the rates of hematologic toxicities between the decitabine-venetoclax and azacitidine-venetoclax cohorts.

We then analyzed the grade three or higher non-hematologic toxicities. Neutropenic fever was the most common (40.0%), followed by infection (38.2%) and hypotension (9.1%). Severe nausea, vomiting, and diarrhea each occurred at a rate of 3.6%. There were no significant differences in rates of non-hematologic toxicities between hypomethylating backbones.

Next, we analyzed the rates and causes of death. The rate of death within 30 days was 8.8%, and the rate of death within 60 days was 21.1%. There were no significant differences in the rates of death with respect to the hypomethylating backbone within 30 days (*p* = 0.639) or 60 days (*p* = 0.335). The cause of death was known in 40 (70.2%) patients. Thirty-one (77.5%) of 40 patients died from relapsed or refractory disease; in contrast, 15.0% died from organ failure, 12.5% from infection, and 5.0% from hemorrhage, as depicted in Figure 2B. Death due to underlying disease was significantly higher than death from any other cause (*p* < 0.0001).

### 3.3 Response

Fifty of 57 patients were evaluable for response. In the overall cohort, ten (20.0%) patients achieved CR, and ten (20.0%) achieved CRi. No patients achieved CRh. Nine (18.0%) patients had MLFS as the best response. The composite complete remission rate (CCR; CR + CRi) was 40.0% (95% CI, 27.6 to 53.8). The response data are presented in Table 3.

Next, we analyzed the responses with respect to the hypomethylating agent backbone. The CCR rate of decitabine-venetoclax was 33.3% (95% CI, 19.2 to 51.2), compared to 50.0% (95% CI, 29.9 to 70.1) for azacitidine-venetoclax; there were no significant differences in response between these cohorts (*p* = 0.258). We then analyzed responses after stratifying patients to the ELN 2022 risk categories. The CCR rate was 37.5% (95% CI, 13.7 to 69.4) for the favorable risk category, 36.4% (95% CI, 15.2 to 64.6) for intermediate risk, and 41.9% (95% CI, 26.4 to 59.2) for adverse risk. There were no significant differences in response between the ELN 2022 risk cohorts (*p* > 0.999). There were also no significant differences in response rates with respect to the hypomethylating agent backbone after stratifying by ELN 2022 risk categories.

We performed subgroup analyses to observe the responses after stratifying by prior treatment. There were no significant differences in response rates between relapsed disease and refractory disease (*p* = 0.572). While response rates numerically favored an azacitidine backbone for both relapsed and refractory disease, there were no significant differences in response rates between the hypomethylating agents. Additionally, there was no significant difference in response rates with respect to prior hypomethylating agent exposure before the initiation of venetoclax-based combination therapy (*p* = 0.450). The CCR rate was 33.3% in patients that underwent a stem cell transplant prior to venetoclax exposure and 40.9% in those that did not, which was not significantly different (*p* > 0.999).

We then analyzed responses in subgroups with specific disease phenotypes and molecular profiles. Patients with monocytic differentiation had a CCR rate of 22.2%, compared to 43.9% without monocytic differentiation (*p* = 0.285). The CCR rate was 50.0% in patients with prior MDS or AML-MRC, compared with 36.8% in *de novo* AML (*p* = 0.506). In patients that achieved a response to venetoclax and a hypomethylating agent and subsequently relapsed, the most common mutations at the time of relapse were in *CBL*, *FLT3*, or *TP53* — which represented 46.2% of new mutations after venetoclax failure. Patients who did not respond to venetoclax-based combination therapy showed significant enrichment in *NRAS*, *KRAS*, and *FLT3*-ITD, independent of mutated *TP53* (*p* = 0.032). Conversely, patients who responded to venetoclax and a hypomethylating agent were significantly enriched in *NPM1*, *IDH1*, and *IDH2* without co-mutations in *NRAS*, *KRAS*, or *FLT3*-ITD (*p* = 0.020).

### 3.4 Survival

**Fig. 3:**
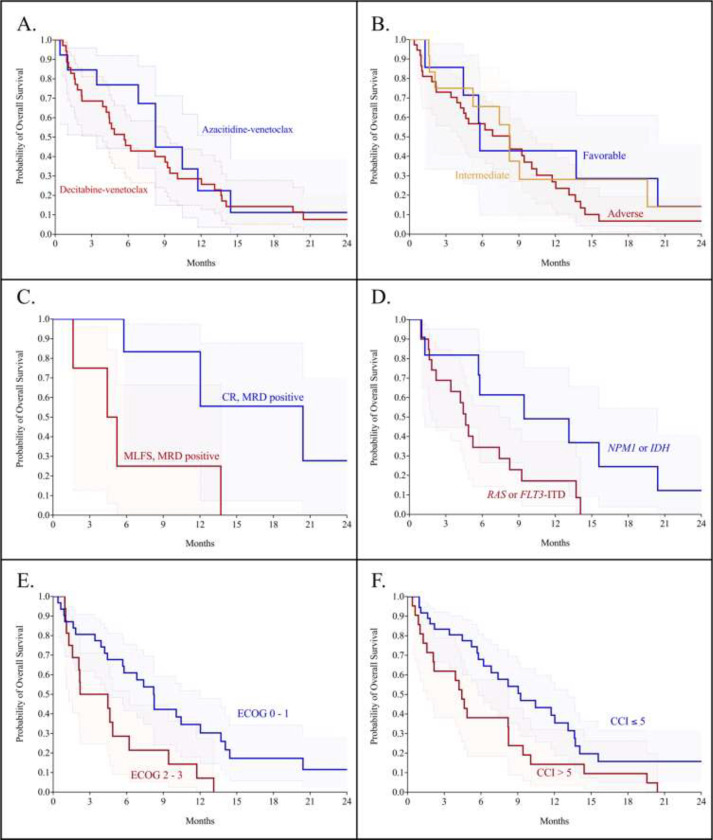
Overall survival in cohorts of relapsed or refractory patients treated with venetoclax and a hypomethylating agent. A. Overall survival of azacitidine-venetoclax was 8.2 months, compared to decitabine-venetoclax at 5.7 months (*p* = 0.425). B. Overall survival of patients stratified by ELN 2022 risk category, with no significant differences between groups (*p* = 0.618). C. Patients in MRD-positive CR had an overall survival of 20.4 months, compared to 4.3 months for MRD-positive MLFS (*p* = 0.035). D. Patients with *NPM1* or *IDH* mutations had an overall survival of 9.4 months, compared to 4.6 months for *RAS* or *FLT3*-ITD mutations (*p* = 0.026). E. Patients with ECOG scores 0 – 1 had an overall survival of 8.2 months compared to 3.3 months for ECOG scores of 2 – 3 (*p* = 0.009). F. A CCI score threshold of 5 or less is associated with superior survival at 9.2 months compared to scores of less than 5 at 4.4 months (*p* = 0.018)

Survival data were available for all 57 patients and are presented in Table 4. The median overall survival of the entire cohort was 8.2 months. Decitabine-venetoclax was associated with a non-significantly shorter overall survival at 5.7 months compared to azacitidine-venetoclax at 8.3 months (*p* = 0.425), as shown in Figure 3A. The progression-free survival of the overall cohort was 4.6 months: when analyzed with respect to the hypomethylating agent, the progression-free survival of decitabine-venetoclax was 4.0 months compared to 5.6 months with azacitidine-venetoclax (*p* = 0.334).

Next, we investigated the impact of the procession to allogeneic stem cell transplant on survival. The median overall survival of patients that proceeded to transplant following treatment with venetoclax was non-significantly longer at 12.0 months, compared to 6.2 months for patients that forewent transplant (*p* = 0.125). Since all of the patients that proceeded to stem cell transplant did so in either CR or MLFS, we performed an ad hoc analysis to investigate the prognostic benefit of MRD-positive patients in CR or MLFS. We found that patients that achieved an MRD-positive CR had significantly superior overall survival at 20.4 months compared to MRD-positive MFLS at 4.3 months (*p* = 0.035, Figure 3C). While a higher proportion of patients in MRD-positive CR proceeded to transplant, there was no significant difference in the receipt of transplant between the MRD-positive CR and MLFS cohorts (*p* = 0.200). We then compared patients who achieved MRD-positive MLFS and also did not undergo transplant to those that were refractory to venetoclax; there was no difference in overall survival between these cohorts (*p* = 0.480).

We subsequently analyzed the overall survival in patients stratified by the ELN 2022 risk categories. The median overall survival was 5.8 months for favorable risk, 8.2 months for intermediate risk, and 8.3 months for the adverse risk category, shown in Figure 3B. There were no significant differences in the overall survival between any ELN 2022 risk cohorts (*p* = 0.618). Additionally, there was no difference in the overall survival when stratified by the ELN 2022 risk categories with respect to the hypomethylating agent backbone. To investigate this disparity, we analyzed the impact of *IDH* and *RAS* mutations in addition to existing mutations accounted for in ELN 2022 on overall survival. We discovered that *IDH* or *NPM1* mutations are associated with a significant survival benefit at 9.4 months compared with at 4.6 months for mutated *NRAS*, *KRAS*, or *FLT3*-ITD (*p* = 0.026, Figure 3D).

We then analyzed survival by patient fitness and comorbidities. Patients with an ECOG score of 0 to 1 had significantly superior overall survival at 8.2 months compared to 3.3 months for patients with an ECOG score of 2 to 3 (*p* = 0.009, Figure 3E). To assess the impact of comorbidities on survival, we performed consecutive survival analyses starting with a Charlson comorbidity index (CCI) score of 3 and continued in increasing CCI score increments until a significant threshold was reached. We discovered that a CCI score threshold of 5 identifies patients at elevated risk of early death. Patients with a CCI score of greater than or equal to 5 had an overall survival of 4.4 months, and those with a score of less than 5 had a median survival of 9.2 months (*p* = 0.018, Figure 3F).

## 4. Outcomes after Venetoclax Failure

**Fig. 4:**
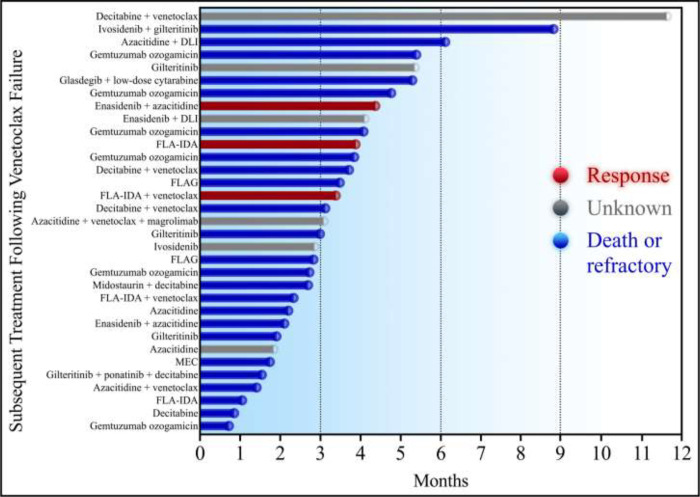
Swimmer plot of each subsequent treatment following venetoclax failure. DLI: donor lymphocyte infusion. FLA-IDA: fludarabine, cytarabine, and idarubicin. MEC: mitoxantrone, etoposide, and cytarabine. Responders are colored red, patients that died or did not respond are colored blue, and patients with an unknown response are colored grey

Next, we analyzed the efficacy of 34 regimens following venetoclax failure and stratified them by therapeutic class, presented in Table 5. Patients who received an IDH inhibitor — ivosidenib or enasidenib — with or without azacitidine or donor lymphocyte infusion, were associated with a non-significantly longer median overall survival at 4.1 months. There was no significant difference in response rates between intensive cytarabine-based chemotherapy and lower-intensity therapies (*p* = 0.145). Similarly, when analyzed by treatment phase, there was no difference in the median overall survival with intensive chemotherapy versus lower-intensity strategies (2.8 versus 3.1 months, *p* = 0.090). Overall, there were no significant differences between any treatment cohorts after venetoclax failure. A swimmer plot of each salvage treatment phase is depicted in Figure 4.

## 5. Discussion

We present several novel findings in relapsed or refractory AML treated with venetoclax and decitabine or azacitidine in the context of ELN 2022. While we acknowledge the limitations of retrospective studies, we emphasize multiple novel findings with regard to toxicity, response, survival, and selection of therapeutic candidates.

Toxicities of venetoclax-based combinations with respect to the hypomethylating agent are an area of interest where little is known outside of clinical trials. In the relapsed or refractory setting, we observed no significant differences in toxicity between decitabine or azacitidine backbones. These findings contrast with the front-line setting, where we previously reported significantly pronounced thrombocytopenia associated with decitabine [7]. Nevertheless, we highlight that failure of disease control remains the most common cause of death in the relapsed or refractory setting.

Our response rates are lower than those observed in clinical trials, stemming from the larger proportion of patients with increased ECOG and CCI scores. We discovered no significant differences in response or survival when patients were stratified by the ELN 2022 criteria. This suggests that the ELN 2022 revision is less applicable in the relapsed or refractory setting and should be refined for patients treated with lower-intensity venetoclax-based strategies. Building upon this, we found significant enrichment in mutated *IDH* and *NPM1* in responders and *RAS* and *FLT3*-ITD in non-responders. Incorporating these novel molecular categories may lead to an improved response classification schema.

More strikingly, we discovered significantly improved survival of patients with mutated *IDH* and *NPM1* compared to *NRAS*, *KRAS*, or *FLT3*-ITD. These findings partially reflect venetoclax sensitivity or resistance, respectively — although we acknowledge that subsequent treatment options, such as IDH inhibitors, likely do influence these results16. Nevertheless, our group and others have reported exceptional response and survival benefits of lower-intensity venetoclax-based strategies in *IDH*^mut^ AML and the implications of FLT3 and RAS-mediated venetoclax resistance [6, 7]. In the context of evolving treatment paradigms, these data provide evidence that relapsed or refractory AML is not associated with a uniform adverse risk profile. Significant response and survival benefits unique to molecular cohorts apply in the relapsed and refractory setting.

In relapsed or refractory patients treated with venetoclax and a hypomethylating agent, receipt of an allogeneic stem cell transplant was associated with prolonged overall survival approaching significance. Since patients that proceeded to stem cell transplant did so in CR or MLFS, we analyzed the impact of MRD positivity after reaching either of these two responses. We provided evidence of a significant survival benefit for patients in MRD-positive CR compared to MRD-positive MLFS, which was impacted by more patients in MRD-positive CR proceeding to transplant. Additionally, we observed no significant survival difference between MRD-positive MLFS and those refractory to venetoclax. These findings suggest that in the context of lower-intensity venetoclax-based strategies, MRD-positive MLFS behaves similarly to refractory disease.

More importantly, the careful selection of treatment candidates in the relapsed or refractory setting needs to be improved. We identified two parameters to refine the selection of patients treated with venetoclax and a hypomethylating agent outside clinical trials. First, we demonstrated a significant survival disparity between ECOG scores of 0 to 1 and scores of 2 to 3, highlighting the importance of careful assessment of performance status before initiating therapy. Second, the CCI score provides a convenient and clinically meaningful parameter to assess treatment candidates. We previously reported that a CCI score threshold of 7 identified high-risk patients treated with venetoclax and a hypomethylating agent in the front-line setting [7]. We hypothesized that a lower score threshold would apply to the relapsed or refractory setting due to the burden and complications of multiple treatment courses. Indeed, we discovered that a CCI score threshold of 5 identifies patients at a higher risk of death. We provide evidence that the ECOG and CCI scores refine treatment candidates for lower-intensity venetoclax-based strategies outside clinical trials.

There are few reports evaluating subsequent therapies after venetoclax failure. We provide an assessment of all subsequent treatment phases following venetoclax failure. While ivosidenib or enasidenib were associated with the longest overall survival, there were no significant differences between groups — even between intensive multi-agent chemotherapy and targeted therapies. These findings highlight the need for novel agents and combinations, prospective trial designs evaluating sequential therapies, and analyses of therapeutic sequencing outside of clinical trials.

In summary, we analyzed the performance of venetoclax with decitabine or azacitidine in relapsed or refractory AML under the ELN 2022 guidelines. We emphasize three novel findings. First, we demonstrate that the ELN 2022 revision is not optimized for the relapsed setting or for patients treated with lower-intensity venetoclax-based strategies: further refinement is needed. Second, we show unique response and survival benefits for patients with mutated *NPM1* and *IDH*; in contrast, *NRAS*, *KRAS*, and *FLT3*-ITD are associated with inferior response and survival. Third, we demonstrate that a CCI score threshold of 5 is a clinically useful adjunct to improving the selection of therapy candidates.

## Figures and Tables

**Table 1:** Baseline characteristics of patients treated with venetoclax and decitabine or azacitidine. There were no statistical differences between groups in any of the subcategories

Baseline Characteristics			
Characteristic	All patients (N = 57)	Decitabine-venetoclax (N = 35)	Azacitidine-venetoclax (N = 22)
Male sex – no. (%)	28 (49.1)	17 (48.6)	11 (50.0)
Age at diagnosis – yr.			
Median	60	62	59
Range	23 – 81	23 – 81	23 – 76
Race – no. (%)^A^			
Black	12 (21.4)	10 (29.4)	2 (9.1)
White	43 (76.8)	23 (67.6)	20 (90.9)
Other	1 (1.8)	1 (2.9)	0 (0)
ELN 2022 cytogenetic risk group – no. (%)			
Favorable	8 (14.0)	7 (20.0)	1 (4.5)
Intermediate	12 (21.1)	6 (17.1)	6 (2.3)
Adverse	37 (64.9)	22 (62.9)	15 (68.2)
Molecular aberrations – no. (%)^B^			
*ASXL1*	10 (18.2)	6 (17.6)	4 (19.0)
*BCOR*	3 (5.5)	2 (5.9)	1 (4.8)
*BCR::ABL1*	1 (1.8)	1 (2.9)	0 (0)
*CEBPA* ^biallelic^	1 (1.8)	1 (2.9)	0 (0)
*CEBPA* ^monoallelic^	2 (3.6)	1 (3.7)	1 (4.8)
*DNMT3A*	11 (20.0)	4 (11.8)	7 (33.3)
*FLT3*-ITD	11 (20.0)	6 (17.6)	5 (23.8)
*FLT3*-TKD	9 (16.4)	7 (20.6)	2 (9.5)
*IDH1*	5 (9.1)	4 (11.8)	1 (4.8)
*IDH2*	6 (10.9)	2 (5.9)	4 (19.0)
*KRAS*	6 (10.9)	5 (14.7)	1 (4.8)
*NPM1*	13 (23.6)	9 (26.5)	4 (19.0)
*NRAS*	12 (21.8)	7 (20.6)	5 (23.8)
*RUNX1*	9 (16.4)	7 (20.6)	2 (9.5)
*SF3B1*	1 (1.8)	1 (2.9)	0 (0)
*SRSF2*	5 (9.1)	3 (8.8)	2 (9.5)
*STAG2*	7 (12.7)	4 (11.8)	3 (14.3)
*TP53*	9 (16.4)	7 (20.6)	2 (9.5)
*U2AF1*	3 (5.5)	1 (2.9)	2 (9.5)
*ZRSR2*	4 (7.3)	2 (5.9)	2 (9.5)
AML-MRC – no. (%)^C^	12 (22.6)	8 (25.0)	4 (19.0)
Previously diagnosed MDS – no. (%)	4 (7.0)	3 (8.6)	1 (4.5)
Charlson comorbidity index score			
Median	4	5	4
Range	2 – 14	2 – 14	2 – 7
ECOG at diagnosis			
Median	1	1	1
Range	0 – 3	0 – 3	0 – 3
Prior stem cell transplant – no. (%)	8 (14.0)	3 (8.6)	5 (19.0)
Prior hypomethylating agent – no. (%)	11 (19.3)	6 (17.1)	5 (22.7)
Disease status – no. (%)			
Relapsed	24 (42.1)	13 (37.1)	11 (50.0)
Refractory	33 (57.9)	22 (62.9)	11 (50.0)
Prior lines of therapy			
Median	1	1	1
Range	1 – 7	1 – 4	1 – 7
Number of cycles			
Median	2	2	3
Range	1 – 10	1 – 10	1 – 9

A: Race was known in 56 of 57 patients

B: Fifty-five of 57 patients had NGS evaluable prior to initiation of venetoclax

C: Fifty-three of 57 patients were evaluable for AML-MRC at the time of diagnosis

**Table 2:** Grade 3 or higher toxicities in patients treated with venetoclax and decitabine or azacytidine

Toxicity				
Toxicity Type	Total treatment courses^A^ (N = 55)^B^	Decitabine-venetoclax (N = 34)	Azacitidine-venetoclax (N = 21)	Significance
Hematologic toxicities, grade ≥3 – no. (%)				
Leukopenia	50 (90.9)	31 (91.2)	19 (90.5)	*p* > 0.999
Neutropenia	54 (98.2)	34 (100.0)	20 (95.2)	*p* = 0.382
Lymphocytopenia	43 (78.2)	27 (79.4)	16 (76.2)	*p* > 0.999
Anemia	48 (87.3)	31 (91.2)	17 (81.0)	*p* = 0.408
Thrombocytopenia	50 (90.9)	32 (94.1)	18 (85.7)	*p* = 0.359
Non-hematologic toxicities, grade ≥3 – no. (%)				
Neutropenic fever	22 (40.0)	14 (41.2)	8 (38.1)	*p* > 0.999
Infection	21 (38.2)	13 (38.2)	8 (38.1)	*p* > 0.999
Hypotension	5 (9.1)	3 (8.8)	2 (9.5)	*p* > 0.999
Respiratory failure	4 (7.3)	3 (8.8)	1 (4.8)	*p* > 0.999
Hemorrhage	3 (5.5)	3 (8.8)	0 (0)	*p* = 0.279
Vomiting	2 (3.6)	2 (5.9)	0 (0)	*p* = 0.519
Diarrhea	2 (3.6)	2 (5.9)	0 (0)	*p* = 0.519
Nausea	2 (3.6)	2 (5.9)	0 (0)	p = 0.519
AST elevation	2 (3.6)	1 (2.9)	1 (4.8)	*p* > 0.999
ALT elevation	1 (1.8)	0 (0)	1 (4.8)	*p* = 0.382
DIC	1 (1.8)	1 (2.9)	0 (0)	*p* > 0.999
TLS	1 (1.8)	1 (2.9)	0 (0)	*p* > 0.999
Cardiomyopathy	1 (1.8)	1 (2.9)	0 (0)	*p* > 0.999
Creatinine increased	1 (1.8)	0 (0)	1 (4.8)	*p* = 0.382
Death during induction – no. (%)^C^				
Death within 30 days	5 (8.8)	4 (11.4)	1 (4.5)	*p* = 0.639
Death within 60 days	12 (21.1)	9 (25.7)	3 (13.6)	*p* = 0.335

A: A treatment course was defined as the initiation of venetoclax with decitabine or azacitidine, continued as maintenance with the same hypomethylating agent backbone in consecutive cycles

B: Fifty-three of 57 patients had toxicity data available for analysis. One patient from the decitabine and azacitidine cohorts switched the hypomethylating agent during maintenance, accounting for one additional patient to each cohort

C: Rates of death during induction were known in all 57 patients

**Table 3:** Response of patients treated with venetoclax and decitabine or azacytidine

Response				
Response Category	All patients (N = 50)^A^	Decitabine-venetoclax (N = 30)	Azacitidine-venetoclax (N = 20)	Significance
Complete remission (CR)	10 (20.0)	5 (16.7)	5 (25.0)	*p* = 0.494
Complete remission with incomplete hematologic recovery (CRi)	10 (20.0)	5 (16.7)	5 (25.0)	*p* = 0.494
Composite complete remission (CR + CRi)	20 (40.0)	10 (33.3)	10 (50.0)	*p* = 0.258
Composite complete remission by ELN 2022 cytogenetic risk category – no. (%)				
Favorable^B^	3 (37.5)	3 (42.9)	0 (0)	*p* > 0.999
Intermediate^C^	4 (36.4)	2 (33.3)	2 (40.0)	*p* > 0.999
Adverse^D^	13 (41.9)	5 (29.4)	8 (53.3)	*p* = 0.157
Composite complete remission by disease status – no. (%)				
Relapsed^E^	9 (45.0)	4 (36.4)	5 (55.6)	*p* = 0.653
Refractory^F^	11 (36.7)	6 (31.6)	5 (45.5)	*p* = 0.696
Composite complete remission with respect to hypomethylating agent exposure – no. (%)				
Prior hypomethylating agent^G^	2 (25.0)			
No prior hypomethylating agent^H^	18 (42.9)			

A: Fifty of 57 patients were evaluable for response: 30 in the decitabine cohort and 20 in the azacitidine cohort

B: In the favorable risk category, seven patients in the decitabine cohort and one in the azacitidine cohort were evaluable

C: In the intermediate risk category, six patients in the decitabine cohort and five in the azacitidine cohort were evaluable

D: In the adverse risk category, 17 patients in the decitabine cohort and 14 in the azacitidine cohort were evaluable

E. In the relapsed setting, 11 patients in the decitabine cohort and nine in the azacitidine cohort were evaluable

F. In the refractory setting, 19 patients in the decitabine cohort and 11 in the azacitidine cohort were evaluable

G. Response was evaluable in eight of 11 patients exposed to a hypomethylating agent

H. Response was evaluable in 42 of 46 patients with no prior hypomethylating agent exposure

**Table 4:** Survival of patients treated with venetoclax and decitabine or azacytidine

Survival				
	All patients (N = 57)	Decitabine-venetoclax (N = 35)	Azacitidine-venetoclax (N = 22)	Significance
Median overall survival – m.	8.2 m.	5.7 m.	8.3 m.	*p* = 0.425
Progression-free survival – m.	4.6 m.	4.0 m.	5.6 m.	*p* = 0.334
Overall survival by ELN 2022 cytogenetic risk category – no. (%)				
Favorable^A^	5.8 m.	5.8 m.	—	—
Intermediate	8.2 m.	8.6 m.	7.4 m.	*p* = 0.702
Adverse	8.3 m.	4.5 m.	10.5 m.	*p* = 0.086

A: The overall survival for the favorable risk category is undefined in the azacitidine-venetoclax cohort (N = 1)

**Table 5:** Overall survival of reinduction regimens following venetoclax failure

Survival After Venetoclax Failure	
Reinduction regimen — (no.)	Median overall survival – m.
Ivosidenib or enasidenib +/− azacitidine (5)	4.1 m.
Gemtuzumab ozogamicin (6)	3.7 m.
Venetoclax + decitabine or azacitidine (5)	3.1 m.
FLAG-based (6)	3.1 m.
FLT3 inhibitor (6)	2.8 m.
Decitabine or azacitidine (4)	2.0 m.

## Data Availability

The data that support the findings of this study are available from the corresponding author upon reasonable request. This dataset is part of the Project ERIS database at VCU Massey Cancer Center.
